# The Effects of AI-Supported Autonomous Irrigation Systems on Water Efficiency and Plant Quality: A Case Study of *Geranium psilostemon* Ledeb

**DOI:** 10.3390/plants14050770

**Published:** 2025-03-03

**Authors:** Gülcay Ercan Oğuztürk, Caner Murat, Meryem Yurtseven, Türker Oğuztürk

**Affiliations:** 1 Department of Landscape Architecture, Recep Tayyip Erdoğan University, Rize 53020, Türkiye; meryem_yurtseven20@erdogan.edu.tr (M.Y.); turker.oguzturk@erdogan.edu.tr (T.O.); 2 Department of Electrical and Electronics Engineering, Recep Tayyip Erdoğan University, Rize 53020, Türkiye; caner.murat@erdogan.edu.tr

**Keywords:** artificial intelligence, AI-supported irrigation, water efficiency, plant cultivation, rize

## Abstract

This study investigates the effects of an AI-supported irrigation system on the production of natural plant species and irrigation efficiency at Rize Recep Tayyip Erdoğan University. To enhance water resource efficiency while utilizing Turkey’s rich plant diversity, *Geranium psilostemon* Ledeb. (Black-Eyed Crane’s-Bill) was selected for cultivation. The research includes adaptation trials and growth monitoring of this perennial taxon, which naturally grows at an altitude of 2000 m. The experiments were conducted in two different environments: one utilizing an AI-supported irrigation system and the other relying on manual irrigation. The findings reveal that AI-supported irrigation systems optimize irrigation strategies, providing a more efficient and effective plant cultivation process compared to manual irrigation. The AI-supported irrigation system continuously monitors air and soil moisture levels, ensuring optimal irrigation conditions and instant adaptation to seasonal variations. This innovative approach minimizes water losses while preventing soil salinization, thereby offering a significant solution for sustainable agricultural practices. In conclusion, this study demonstrates that natural plant species can be effectively cultivated using AI-supported irrigation systems and that these systems hold great potential for water conservation and ecological balance. These findings present a crucial step toward developing effective solutions for global water challenges and promoting sustainable landscape and agricultural practices.

## 1. Introduction

The rapid increases in urbanization, climate change, and depletion of water resources have made the sustainable management of natural resources a necessity. Efficient and effective use of water resources is crucial for addressing global water scarcity and maintaining ecological balance [1,2,3,4,5,6,7]. In this context, AI-supported (Artificial Intelligence) smart irrigation systems offer innovative solutions for the sustainable management of water resources [8,9,10]. These systems play a vital role in analyzing sensor data to determine irrigation timing, quantity, and optimal conditions based on plant needs. By leveraging sensor-based monitoring, real-time data analysis, and machine learning algorithms, AI-driven irrigation systems automate irrigation processes, reduce dependence on human intervention, and minimize water loss [11,12,13,14].

AI-supported irrigation systems not only optimize water usage in agricultural and landscape applications but also enhance plant growth and quality by continuously monitoring dynamic parameters such as soil moisture levels, weather conditions, and plant growth cycles [15,16,17,18]. However, the integration of such systems with natural plant species and their impact on water efficiency has not been sufficiently explored in the literature [19,20,21]. Previous studies have primarily focused on agricultural crops, overlooking the potential of native species in landscape applications. This gap highlights the need for further research on the contributions of AI-driven systems to ecological sustainability and the conservation of natural resources.

Recent studies emphasize the potential of AI-driven irrigation systems in optimizing water use, and enhancing plant health [22] showed that real-time water monitoring using AI significantly improved water use efficiency and supported environmental sustainability. Ref. [23] reported enhanced water conservation and productivity in water-scarce regions through low-cost IoT-based irrigation systems, while [24] demonstrated that AI-supported systems optimized soil moisture management, resulting in 30–40% greater water efficiency compared to manual irrigation. Ref. [25] found that intelligent fertigation systems effectively managed nutrients under variable water quality conditions, thereby enhancing plant health, and [26] reported that robotic and AI-assisted autonomous agricultural systems reduced plant stress and increased growth rates. These studies collectively highlight the potential of AI-driven irrigation systems to enhance water efficiency, reduce plant stress, and promote growth consistency, particularly under dynamic environmental conditions.

Turkey’s rich biodiversity presents significant potential for supporting sustainable agriculture and landscape applications through the use of native plant species. However, there is a need for comprehensive studies on the integration of local plant species with AI-supported irrigation systems and their impact on water efficiency and plant quality [27,28]. Specifically, native species such as *Geranium psilostemon* Ledeb. (Black-Eyed Crane’s-Bill), which naturally grows at an altitude of 2000 m and thrives under varying climatic conditions, can serve as ideal models for water-saving and sustainable solutions in landscape applications.

This study examines the cultivation of *Geranium psilostemon* Ledeb. using AI-supported irrigation systems, comparing it with manual irrigation methods. It hypothesizes that AI-supported irrigation systems will improve water efficiency and provide better growth and health indicators for *Geranium psilostemon* Ledeb. compared to manual irrigation. This hypothesis is based on the premise that AI-supported irrigation systems can more precisely manage soil moisture, optimize water usage, and reduce plant stress through adaptive learning algorithms.

This hypothesis is supported by findings from [22], who demonstrated that autonomous real-time water monitoring systems increased water efficiency and promoted environmental sustainability. Similarly, [23] reported that IoT-based irrigation systems enhanced water conservation, particularly in water-scarce regions. Ref. [24] confirmed that AI-supported systems optimized soil moisture management, achieving up to 40% greater water efficiency compared to manual irrigation, and [25] observed that intelligent fertigation systems effectively managed nutrients under varying water quality conditions, enhancing plant health. Additionally, [26] demonstrated that robotic and AI-assisted agricultural systems improved growth rates and reduced plant stress.

Confirming this hypothesis would provide valuable scientific and practical insights into water conservation and sustainable agricultural practices. Therefore, this study aims to evaluate the effects of AI-supported irrigation on water efficiency, plant growth, and quality using *Geranium psilostemon* Ledeb. as a model species. The findings are expected to contribute to the broader understanding of AI-supported irrigation systems and their potential applications in sustainable agriculture and landscape management.

## 2. Results

### 2.1. Germination and Growth Findings

The study was conducted in two phases, referred to as first planting and second planting. Monthly measurements were performed during each planting period, and all germinated leaves in the pots were systematically recorded in detail.

**Figure 1:**
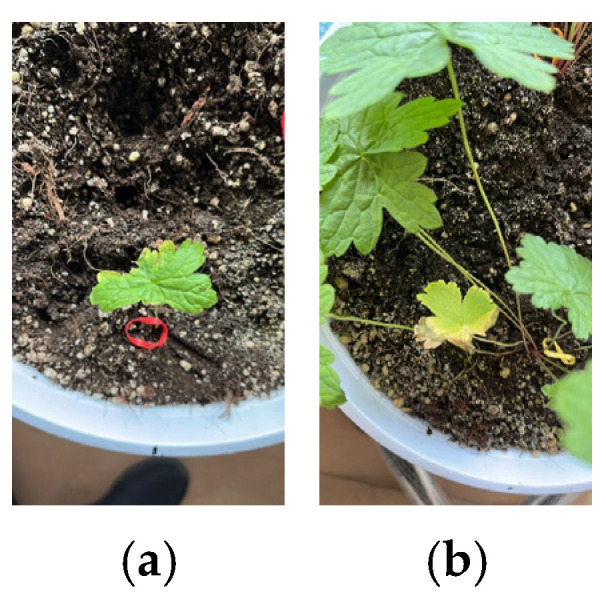
(**a**) Yellowing plant, (**b**) plant exhibiting yellowing and desiccation.

**Figure 2:**
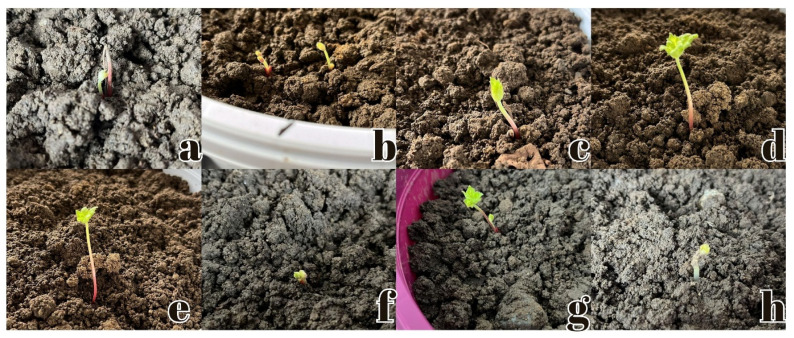
Visual documentation of measurements conducted on 6 December 2023. (**a**) Initial seed cracking and emergence of the embryo above the soil surface, (**b**) Emergence of the primary root and shoot, growing towards the soil surface, (**c**) Budding of the first leaves and elongation of the stem, (**d**) Leaf unfolding in preparation for photosynthesis, (**e**) Elongation of the stem and continued leaf growth, (**f**) Early germination stage with initial leaf development, (**g**) Growth of young leaves and strengthening of the stem, (**h**) Final stages of germination with leaf expansion and upright growth of the seedling.

**Figure 3:**
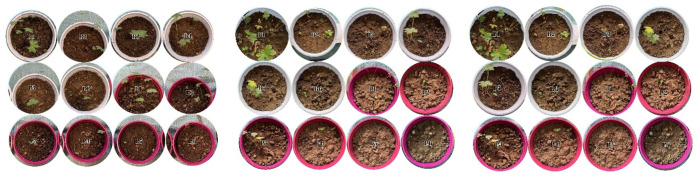
Visual documentation of measurements conducted on 5 January 2024, 2 February 2024, and 1 March 2024.

1. Planting Phase: Initially, the humidity level was set within the range of 43–45. However, due to an increase in ambient temperature, P1 (Plant 2), P4 (Plant 1), and P5 (Plant 1) experienced desiccation. In response to this observation, the lower and upper humidity thresholds were revised to 50–55. Additionally, B5 (Plant 1) exhibited mild yellowing, while B5 (Plant 3) showed signs of progressive yellowing and desiccation (Figure 1). Visual documentation of the measurements conducted on 6 December 2023 is presented in Figure 2. The visual documentation of the measurements conducted on 5 January 2024, 2 February 2024, and 1 March 2024 is provided in Figure 3.

**Figure 4:**
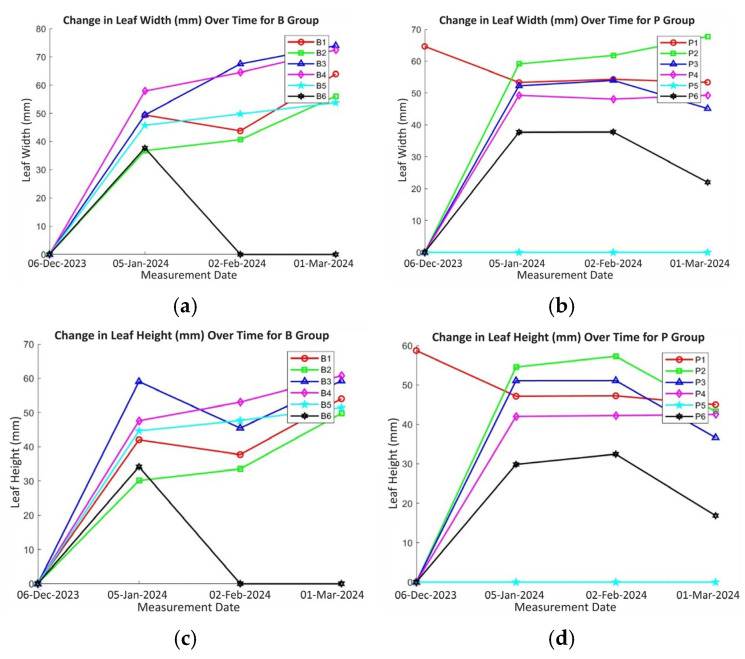

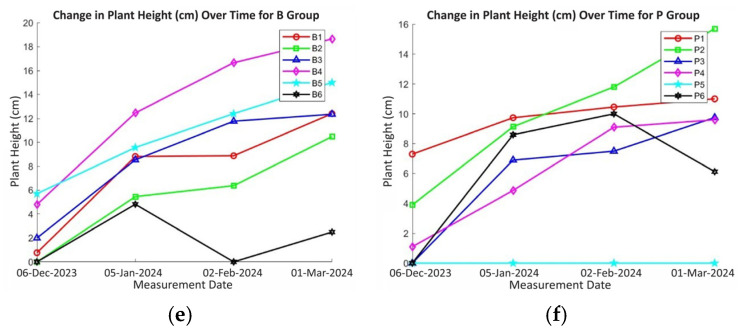
Leaf width vs. date for (**a**) Group B and, (**b**) Group P, (**c**) leaf height vs. date for Group B and, (**d**) Group P, (**e**) plant height vs. date for Group B and, (**f**) Group P in first cultivation.

Figure 4a–f present the results of an experiment investigating the growth of plants under two watering regimes: autonomous watering (Group B) and manual watering (Group P). Six flowerpots were assigned to each group. To provide context for the growth data, soil moisture content and pH were monitored in each pot at three time points: 5 January 2024, 2 February 2024, and 1 March 2024. In Group B, soil moisture in B1 varied from 67.4% to 55.4% with pH ranging from 6.35 to 7.01. B2 exhibited soil moisture fluctuations between 60.7% and 64%, and pH ranged from 5.62 to 7.9. B3 showed soil moisture ranging from 72.7% to 65% with pH from 5.72 to 7.8. B4’s soil moisture varied from 49.1% to 79.9% with pH from 5.8 to 6.5. B5 displayed soil moisture between 42.3% and 60.7% with pH from 6.54 to 5.83. B6 exhibited soil moisture between 51.7% and 62.1% with pH from 6.1 to 7.4. In Group P, P1’s soil moisture varied from 52.2% to 59.5% with pH from 6.1 to 7.5. P2’s soil moisture ranged from 68.4% to 73.4% with pH from 6.42 to 7.35. P3’s soil moisture ranged from 73.1% to 74.9% with pH from 6.15 to 8. P4’s soil moisture varied from 33% to 71% with pH from 6.18 to 7.9. P5’s soil moisture ranged from 69.6% to 65.5% with pH from 5.79 to 6.5. P6’s soil moisture varied from 57.1% to 57% with pH from 5.94 to 6.73. This information provides crucial context for understanding the plant growth patterns observed in each group under the respective watering regimes.

Figure 4a illustrates the growth trajectories of six individual plants (B1–B6) under an AI-supported irrigation system from 6 December 2023 to 1 March 2024. A general upward trend in leaf width is evident across most plants, indicative of effective water management. Plant B2 consistently exhibited the largest leaf width, suggesting optimal water and nutrient uptake. B1 and B3 demonstrated steady growth, while B4 exhibited a significant increase in leaf width, potentially due to a period of rapid growth or an increase in water availability. B5 maintained a relatively stable leaf width, suggesting consistent water availability and a balanced growth rate. The anomalous behavior of plant B6, with a sharp increase followed by an abrupt decline, suggests potential issues such as temporary stress due to brief periods of overwatering or underwatering, environmental stressors, or localized soil issues affecting root development and water uptake. In contrast, Figure 4b compares the growth of two groups of plants: Group B, irrigated by an AI-supported irrigation, and Group P, subjected to manual watering. Group B demonstrated more consistent and robust growth across the majority of plants compared to Group P. The AI-supported irrigation system in Group B appears to have optimized water delivery for each plant, leading to higher leaf widths in B2 and steady growth in B1 and B3. While an anomaly in B6 suggests a potential area for improvement in the AI model, the overall trend in Group B indicates effective water management. Group P exhibited greater variability in growth patterns, suggesting inconsistencies in manual watering that may have resulted in instances of overwatering or underwatering. This comparison highlights the potential advantages of AI-supported irrigation systems, including consistent and tailored water delivery, optimized water usage, and reduced labor requirements, which collectively contribute to improved plant growth and yield compared to manual watering practices. Although the controlled environment minimizes some factors like spacing and soil type, variations in root development, soil conditions, light availability, and the activity of soil microorganisms likely contribute to the observed differences in plant growth.

Figure 4c,d illustrate the leaf height of two groups of plants over a period from 6 December 2023 to 1 March 2024. Figure 4c depicts the leaf height of six plants (B1–B6) under an AI-supported irrigation system, revealing a general upward trend in leaf height, indicative of overall growth. Plant B2 exhibits the highest leaf height, suggesting optimal growth conditions and likely efficient water and nutrient uptake. B1 and B3 demonstrate steady growth, while B4 exhibits a significant increase in leaf height, potentially due to a period of rapid growth or an increase in water availability. B5 maintains a relatively stable leaf height, suggesting consistent water availability and a balanced growth rate. Notably, plant B6 exhibits an anomalous pattern with a sharp increase followed by a sharp decline, suggesting potential issues like overwatering or localized environmental stress affecting root development and water uptake. In contrast, Figure 4d, depicting leaf height in Group P, which was subjected to manual watering, reveals more variable growth patterns compared to the AI-supported irrigation group in Figure 4c. This variability suggests that manual watering may not be as consistent or responsive to individual plant needs as the AI-supported irrigation system. Some plants in Group P show significant fluctuations in leaf height, indicating potential inconsistencies in water delivery that may have resulted in overwatering or underwatering. These fluctuations in leaf height can be attributed to various factors, including variations in water availability, nutrient uptake, light availability, temperature, and other environmental conditions. Adequate and consistent water supply is crucial for plant growth, while overwatering can lead to root hypoxia and nutrient leaching, hindering growth. Conversely, insufficient water can limit photosynthesis and overall plant development. Other factors such as nutrient availability, light intensity, temperature, and soil conditions also significantly influence plant growth and development.

Figure 4e,f illustrate the plant height of two groups of plants over a period from 6 December 2023 to 1 March 2024. Figure 4e depicts the growth of plants under an AI-supported irrigation system (Group B), revealing a general upward trend in plant height across most plants, indicative of overall growth. Plant B2 exhibits the highest plant height, suggesting optimal growth conditions and likely efficient water and nutrient uptake. B1 and B3 demonstrate steady growth, while B4 exhibits a significant increase in plant height, potentially due to a period of rapid growth or an increase in water availability. B5 maintains a relatively stable plant height, suggesting consistent water availability and a balanced growth rate. Notably, plant B6 displays an anomalous pattern with a sharp increase followed by a sharp decline, suggesting potential issues like overwatering or localized environmental stress affecting root development and water uptake. In contrast, Figure 4f, depicting the growth of plants under manual watering (Group P), reveals more variable growth patterns compared to the AI-supported irrigation group in Figure 4e. This variability suggests that manual watering may not be as consistent or responsive to individual plant needs as the AI-supported irrigation system. Some plants in Group P show significant fluctuations in plant height, indicating potential inconsistencies in water delivery that may have resulted in overwatering or underwatering. These fluctuations in plant height can be attributed to variations in factors such as water availability, nutrient uptake, light availability, temperature, and other environmental conditions. The analysis of Figure 4e,f highlights the potential impact of different watering methods on plant development, with the AI-supported irrigation system in Group B demonstrating more consistent and robust growth compared to manual watering in Group P.

**Figure 5:**
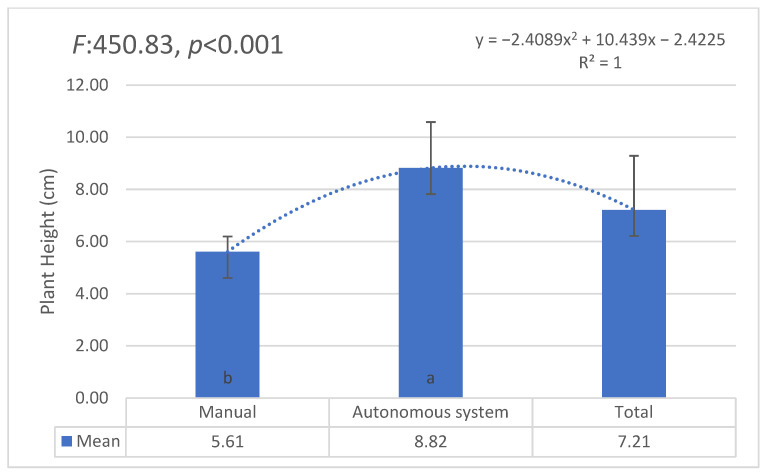
Plant height comparison between manual and AI-supported irrigation systems. Statistically significant differences are indicated by different letters.

The AI-supported system demonstrated more consistent growth across all groups, with a statistically significant difference (F = 6.841, *p* = 0.012), confirming its efficiency in optimizing water use and promoting uniform growth (Figure 5).

**Figure 6:**
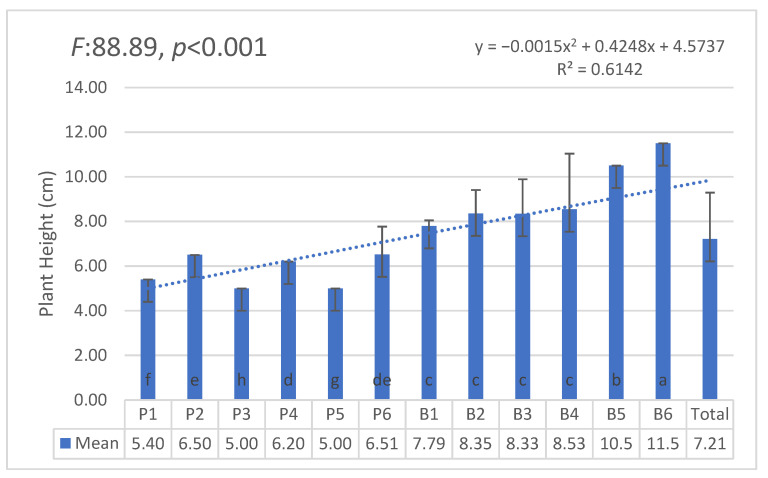
Plant survival rates by irrigation method. Statistically significant differences are indicated by different letters.

The AI-supported irrigation groups showed higher survival rates, whereas the manual irrigation groups exhibited more variability and a higher mortality rate (F = 4.563, *p* = 0.034), highlighting the adaptability and effectiveness of AI-driven irrigation strategies (Figure 6).

### 2.2. Water Consumption and Automation Comparisons

Manual Irrigation (Pink Pots): The manual irrigation method was performed at specific intervals based on user discretion. However, inconsistencies in irrigation frequency and quantity were observed due to variations in ambient temperature. This led to under-irrigation in some periods and over-irrigation in others. Over-irrigation resulted in adverse effects such as root rot, while under-irrigation limited plant growth.

**Figure 7:**
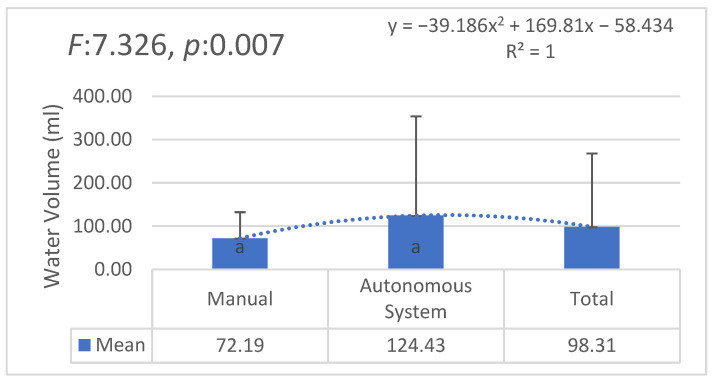
Water consumption comparison between manual and autonomous systems. Statistically significant differences are indicated by different letters.

Figure 7 compares the water consumption between the manual irrigation and the AI-supported autonomous irrigation systems. The results indicate a statistically significant difference (F: 7.326, *p*: 0.007), with the autonomous system using more water on average. However, the variability in water usage is higher in the autonomous system, suggesting a more dynamic adaptation to environmental conditions.

AI-supported irrigation (White Pots): The AI-supported irrigation method was executed using Arduino-based sensors, which triggered irrigation once the soil moisture level reached the lower threshold. This system dynamically adapted to environmental conditions, ensuring optimal water usage and enhancing efficiency in water consumption. By maintaining appropriate moisture levels continuously, it significantly reduced problems related to over-irrigation, such as root rot, and provided a more consistent and controlled irrigation strategy compared to manual irrigation. The AI-supported irrigation system demonstrated superior performance in terms of plant health and water efficiency.

**Figure 8:**
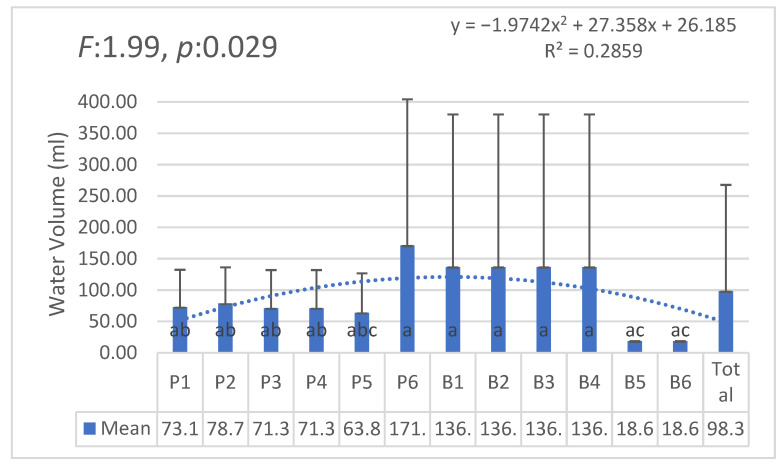
Water consumption by pot groups. Statistically significant differences are indicated by different letters.

Figure 8 shows the variation in water consumption across different pot groups under both irrigation methods. The analysis reveals significant differences (F: 1.99, *p*: 0.029), highlighting the influence of pot position and environmental factors on water usage.

Table 1 presents the water consumption data and percentage reduction for each planting phase and overall average between the AI-supported and manual irrigation systems. The AI-supported irrigation system demonstrated significant water savings, achieving a 76.44% reduction in the first planting and an exceptional 99.5% reduction in the second planting phase. The overall average water consumption reduction was 87.97%, highlighting the efficiency and sustainability potential of the AI-supported irrigation system compared to manual methods.

### 2.3. Second Planting and Soil Revision

After 29 March 2024, surviving rhizomes were transferred to a new experimental setup, and the soil mixture was restructured. The trials were organized into five groups labeled B1–B5 and P1–P5, with each pot containing four rhizomes. Moisture and temperature measurements were monitored at monthly intervals.

The new soil mixture, characterized by a looser texture and improved drainage properties, provided more favorable conditions for plant growth. This adjustment also allowed the sensors used in the AI-supported irrigation system to generate more stable and reliable data, enhancing the accuracy of measurement results.

For the manual irrigation group (pink pots), the rhizomes to be planted were dimensionally evaluated using a caliper, and their weights were determined using a precision scale. The relevant measurements are presented in Table 2.

The caliper measurements and precision scale weights of the rhizomes to be planted in white pots are provided below (Table 3).

The measurements for the second planting were conducted between 5 April 2024 and 3 May 2024 and continued on a monthly basis. The height, count, moisture, and pH values of the plants were recorded.

**Figure 9:**
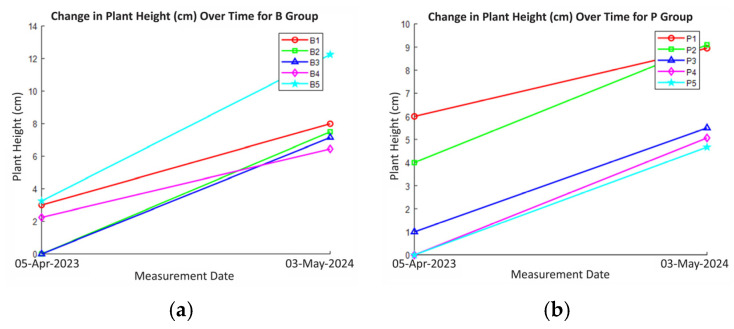

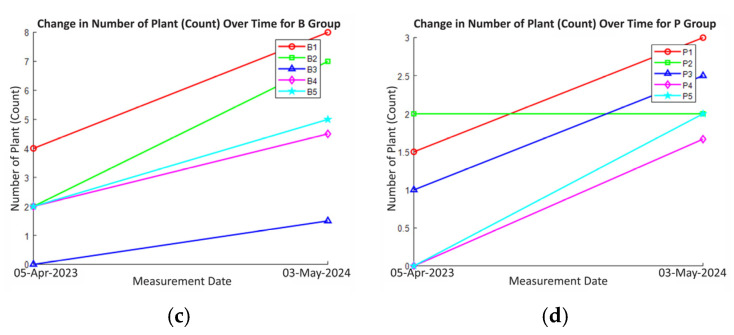
Plant height vs. date for (**a**) Group B and, (**b**) Group P, (**c**) number of plants vs. date for Group B and, (**d**) Group P in second cultivation.

Figure 9a–d illustrate the soil conditions of flowerpots in groups B1–B5 and P1–P5, focusing on humidity and pH changes between 5 April and 3 May 2024. In group B, humidity increased notably in B1 (36.1% to 50.6%), B2 (46.1% to 57%), and B4 (41% to 53.9%), while B3 experienced a slight decrease (48.9% to 46.6%) and B5 remained relatively stable (60% to 57%). Correspondingly, pH levels decreased across all B-group pots, with significant declines in B2 (6.01 to 5.32) and B3 (6.14 to 5.42). In group P, humidity changes were minor, with slight increases in P2 (47.3% to 48.3%) and P5 (47.7% to 50.3%) and slight decreases in P1 (47% to 45.7%) and P3 (49.3% to 48.8%). pH levels increased slightly in P1 (6.02 to 6.1) and P4 (6.07 to 6.04), while the rest exhibited small declines, with P2 dropping from 6.06 to 5.85 and P3 from 6.11 to 5.98. These data highlight temporal variations in soil moisture and acidity across all flowerpots.

Figure 9a,b illustrate the plant height of Group B and Group P, respectively. In Group B, a more consistent and robust upward trend in plant height is observed across most plants, indicating effective growth. Plant B2 consistently exhibits the highest plant height, suggesting optimal growth conditions and likely efficient water and nutrient uptake. B1 and B3 demonstrate steady growth, while B4 exhibits a significant increase in plant height, potentially due to a period of rapid growth or an increase in water availability. B5 maintains a relatively stable plant height. In contrast, Figure 9b shows more variable growth patterns in Group P. Some plants exhibit significant fluctuations in plant height, indicating potential inconsistencies in manual watering, which could lead to overwatering or underwatering, negatively impacting plant growth. Overwatering can lead to root hypoxia, nutrient leaching, and even root rot, hindering growth. Conversely, insufficient water can limit photosynthesis and overall plant development.

Figure 9c,d depict the number of plants over time for Group B and Group P, respectively. Figure 9c shows a relatively stable number of plants in Group B, suggesting that the AI-supported irrigation system did not significantly impact plant mortality. In contrast, Figure 9d shows a slight decrease in the number of plants in Group P over time. This suggests that manual watering may have led to some plant mortality, potentially due to overwatering or underwatering. Overwatering can lead to root rot and suffocation, while underwatering can cause dehydration and wilting, ultimately leading to plant death. Although each plant has the same soil and is watered from the same source, variations in water delivery during manual watering could have contributed to these differences. Additionally, factors like variations in root development, soil compaction, and the activity of soil microorganisms around each plant could also influence water uptake and overall plant health, contributing to the observed differences in plant growth and mortality.

## 3. Materials and Methods

### 3.1. General Characteristics of Geranium psilostemon Ledeb. (Black-Eyed Crane’s-Bill)

**Figure 10:**
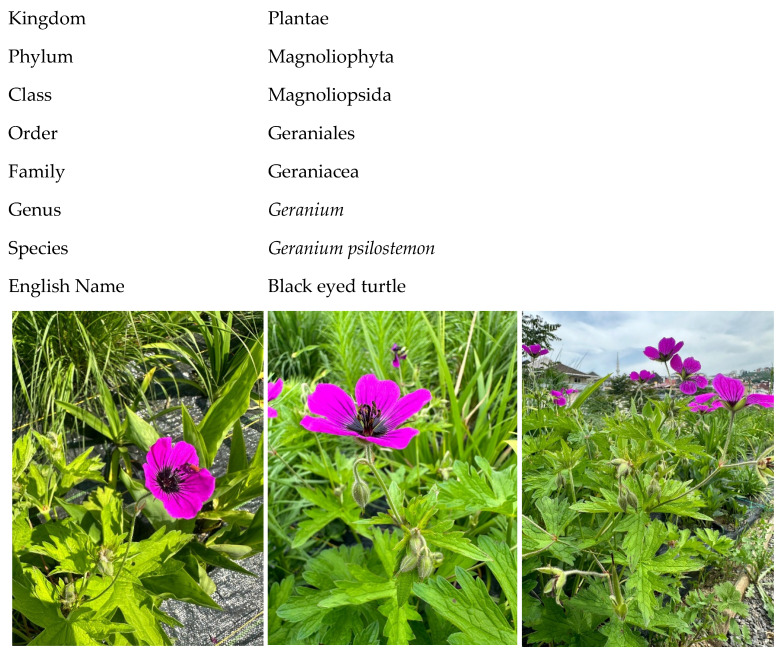
General appearance of *Geranium psilostemon* plant.

*Geranium psilostemon* Ledeb., a hardy and herbaceous perennial plant species belonging to the Geraniaceae family, is commonly known as the “Armenian Crane’s-Bill”. This species is native to Turkey, Armenia, Azerbaijan, and the Russian Federation, where it naturally occurs. The plant grows between 20 and 100 cm in height, has palmately lobed, hairy leaves, and produces magenta-purple flowers (Figure 10).

*Geranium psilostemon* is typically found at altitudes of 1400–2400 m in shrublands, meadows, and forested areas, flowering between June and September. It is valued for both medicinal and ornamental purposes [29,30].

### 3.2. Study Area

**Figure 11:**
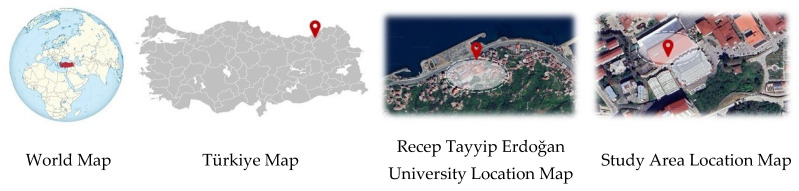
Location map of the study area.

**Figure 12:**
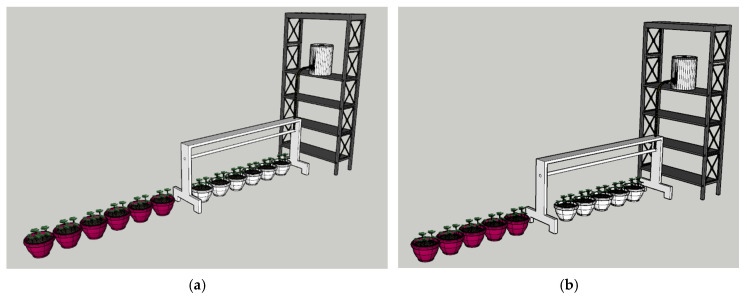
General view of the study area. (**a**) First planting area view, (**b**) second planting area view.

This study was conducted in the laboratory of the Faculty of Engineering and Architecture at Recep Tayyip Erdoğan University Zihni Derin Campus, located in the central district of Rize (Figure 11 and Figure 12).

### 3.3. Experimental Design and Duration

- Experimental Period: Conducted between December and June during the 2023–2024 period.
- Irrigation Experiment: Two different irrigation environments (manual and AI-supported irrigation) were applied to the plants, allowing a comparison in terms of water consumption and efficiency. A total of 12 flowerpots were used in the experiment, with 6 flowerpots allocated for the AI-supported autonomous irrigation group and 6 flowerpots for the manual irrigation group. This design provided sufficient replication to ensure the reliability and validity of the results.
- First and Second Planting: In the first planting, 5 plants were placed in each pot, while in the second planting, 4 plants were used per pot. This approach ensured consistency across all groups while allowing a comparative analysis of plant growth performance under different irrigation regimes. Pink pots represented the manual irrigation system, while white pots represented the AI-supported irrigation system.
- Number of Replications: Each experiment was conducted with six replications per irrigation group (6 flowerpots for AI-supported irrigation and 6 flowerpots for manual irrigation), ensuring statistical reliability and robustness of the results. This design minimized random errors and enhanced the statistical power of the analysis.
- Water Conservation and Growth Observation: As an example of a deficit irrigation approach, the manual and AI-supported irrigation systems were compared based on the plant’s water needs, observing water savings and plant growth performance. Measurements were taken daily to accurately capture temporal variations in soil moisture and plant growth parameters. The average values from the six replications were used in the data analysis to ensure robustness and repeatability of the results.
- Environmental Controls: The experiments were conducted under laboratory conditions to ensure consistency in environmental variables. Temperature was maintained at 23 ± 2 °C and monitored regularly using DHT11 sensors, calibrated weekly with a Testo 605i Thermo-Hygrometer to ensure accuracy. To further ensure the accuracy and reliability of the DHT11 sensors, a weekly calibration process was performed using a Testo 605i Thermo-Hygrometer, which is known for its high precision in humidity and temperature measurements. During the calibration process, the DHT11 sensors were placed alongside the Testo 605i in a controlled environment to compare the readings. The data obtained from the DHT11 sensors were then cross-validated against the Testo 605i values to detect any discrepancies. If deviations beyond ±2% for humidity or ±0.5 °C for temperature were observed, the sensors were recalibrated or replaced. This approach ensured that the DHT11 sensors maintained consistent accuracy throughout the experimental period. The experiments were conducted under controlled laboratory conditions to minimize the influence of seasonal variations. Temperature was maintained at 23 ± 2 °C and humidity levels were consistently regulated using DHT11 sensors, calibrated weekly with a Testo 605i Thermo-Hygrometer. This controlled environment ensured that environmental variables remained constant, allowing the effects of irrigation methods to be accurately evaluated without external climatic influences. LED grow lights provided a 16 h light/8 h dark photoperiod, and light conditions were maintained equally and homogeneously for both groups. All experiments were conducted in the same laboratory environment to ensure consistent environmental conditions, allowing the effects of irrigation methods to be accurately evaluated.

### 3.4. Arduino-Based AI-Supported Irrigation System Design

In the AI-supported irrigation system, an Arduino-based sensor network (DHT11) was utilized. These sensors measure humidity and temperature, enabling automated irrigation by controlling a solenoid valve.

For copper etching, a mixture of ¼ Pehidrol + ¾ Hydrochloric Acid was used, selectively dissolving the copper parts while leaving the ink unaffected. A 0.7 mm hand drill was used to create holes for component placement. Various power sources, such as solar energy or USB output, were used to power the Arduino and valve.

The Arduino MEGA microcontroller was operated at 5V power, while the DHT11 humidity–temperature sensor converted data into a digital signal and transmitted it to the Arduino. The 12V solenoid valve was controlled through a MOSFET circuit to open and close the water flow.

**Figure 13:**
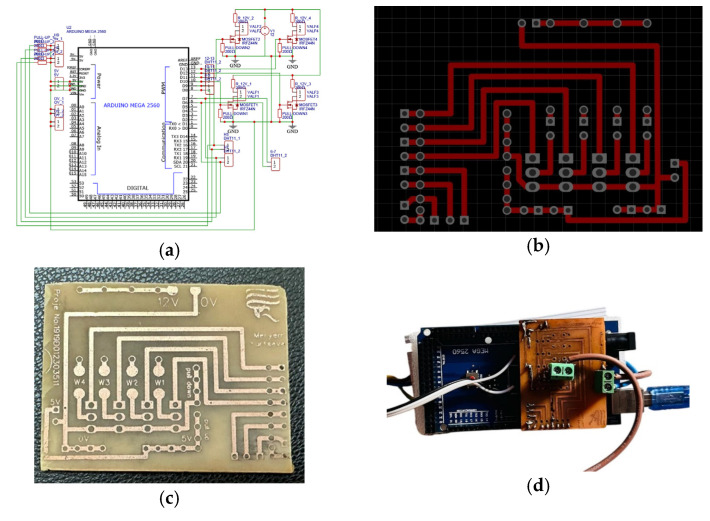
(**a**) Circuit schematic created using EasyEDA software, (**b**) physical layout of the circuit designed with the PCB Studio feature, (**c**) the proposed circuit transferred onto the circuit board, and (**d**) the final assembled version with circuit components.

Using EasyEDA Standard Edition software, the circuit schematic and PCB layout were designed (Figure 13). The PCB studio feature of the same software was used to create the physical layout of the circuit (Figure 13). The layout was then exported as a PDF file for manufacturing.

### 3.5. AI-Supported Autonomous Irrigation Software

The humidity and temperature values obtained from the sensors (float humidity, float temperature) are processed via Arduino, implementing the following logic:

If humidity < 43, a HIGH (1) signal is sent, activating the solenoid valve.

If humidity > 44, a LOW (0) signal is sent, deactivating the solenoid valve.

Once the humidity level reaches the desired threshold, the valve closes, preventing unnecessary water consumption.

The implementation of artificial intelligence (AI) in the irrigation system described in this study utilizes advanced machine learning algorithms to optimize water delivery for plant growth. The AI model used is a Long Short-Term Memory (LSTM) neural network, chosen for its ability to handle sequential data and capture temporal dependencies inherent in environmental factors affecting plant growth.

In the initial phase of model selection, comparative tests were considered with CNN (Convolutional Neural Network), GRU (Gated Recurrent Unit), and RNN (Recurrent Neural Network) models alongside LSTM. However, LSTM was ultimately chosen due to its superior capability in learning sequential patterns and maintaining accuracy over long time intervals, which is critical for time-series data like soil moisture and temperature measurements in irrigation systems. This selection is consistent with previous studies that demonstrated LSTM’s effectiveness in capturing temporal dependencies [31]. Additionally, although CNN achieved the highest accuracy in image-based tasks, its performance in sequential data processing was limited due to its focus on spatial features. Therefore, LSTM was found to be more suitable for this application as it effectively models time-series dependencies, ensuring accurate predictions of irrigation requirements. These findings align with prior research comparing DL architectures [32].

LSTMs, a variant of Recurrent Neural Networks (RNNs), utilize memory cells governed by input, output, and forget gates to selectively retain relevant information over time. The governing equations for the LSTM model are given in Equations (1)–(5) [33].

(1) $$f_{t}=\sigma \left(w_{f}\left[h_{t-1},x_{t}\right]+b_{f}\right)$$

(2) $$i_{t}=\sigma \left(w_{i}\left[h_{t-1},x_{t}\right]+b_{i}\right)$$

(3) $${\tilde{C}}_{t}=\tanh\left(w_{c}\left[h_{t-1},x_{t}\right]+b_{c}\right)$$

(4) $$C_{t}=f_{t}\times C_{t-1}+i_{t}\times {\tilde{C}}_{t}$$

(5) $$o_{t}=\sigma \left(w_{o}\left[h_{t-1},x_{t}\right]+b_{o}\right)$$

(6) $$h_{t}=o_{t}\times \tanh\left(C_{t}\right)$$

The variables used in the LSTM equations represent the following: $f_{t}$ is forget gate activation, $i_{t}$ is input gate activation, ${\tilde{C}}_{t}$ is candidate cell state, $C_{t}$ is cell state), $o_{t}$ is output gate activation, $h_{t}$ is hidden state, $x_{t}$ is current input, *h_t_*_−1_ is previous hidden state, σ is sigmoid activation function, tanh is hyperbolic tangent activation function, $w_{f}$, $w_{i}$ $w_{c}$, $w_{o}$ are weight matrices, and $b_{f}$, $b_{i}$, $b_{c}$, $b_{o}$ are Bias vectors. These equations enable the LSTM to selectively remember and forget information from past inputs, making it a powerful tool for modeling complex sequential data. The core innovation of LSTMs lies in their inclusion of “gates”—mechanisms that control the flow of information into and out of the memory cell. These gates, namely the forget gate, input gate, and output gate, regulate the information that is stored, discarded, and used for the current prediction. By selectively remembering and forgetting information, LSTMs can effectively learn complex patterns and relationships within sequential data, even over extended time intervals.

The training process of LSTM involves adjusting the model’s parameters, including the weights and biases of the gates, to minimize the difference between the predicted output and the actual target values.

To further optimize the model’s performance and generalization capability, Bayesian optimization was used to tune the following hyperparameters: Learning Rate (0.001 to 0.01) to achieve optimal accuracy, Batch Size (32, 64, and 128) to balance computational efficiency and model stability, and Number of LSTM Units (50 to 200) to effectively capture temporal dependencies. A 5-fold cross-validation (k = 5) was applied to evaluate generalization performance, and Bayesian optimization minimized validation loss during each fold. The Adam optimizer was chosen for its computational efficiency. Model performance was assessed using RMSE and MAE metrics. The implementation was conducted using TensorFlow and Keras, ensuring reproducibility.

To validate the predictive performance of the AI model, a 5-fold cross-validation strategy was implemented. In this approach, the dataset was randomly divided into five equal parts, where four parts were used for training and one part for testing in each iteration. This process was repeated five times, ensuring that each subset was used as a test set once. The average accuracy and error rates from the five iterations were calculated to provide a robust evaluation of the model’s generalization performance. This validation strategy minimized overfitting by ensuring that the model was exposed to different subsets of the data during training and testing, enhancing its ability to generalize to unseen data. The results from the 5-fold cross-validation demonstrated consistent accuracy across all folds, confirming the reliability and robustness of the AI-supported irrigation model.

This is typically achieved through backpropagation, an iterative optimization algorithm. Once trained, the LSTM model can be used to make predictions on new, unseen data, such as translating a sentence, generating text, or forecasting future values in a time series. The model was trained using a comprehensive dataset generated through 72 unique measurements collected from the six flowerpots in each group over a three-month period. Measurements included soil moisture content, pH levels, ambient temperature, relative humidity, and light intensity. The temporal resolution of the data was set to daily, ensuring sufficient granularity for capturing short-term fluctuations while maintaining computational feasibility. Each pot’s historical data served as an independent series for training the LSTM, allowing the model to account for pot-specific variations in soil and plant conditions.

Before training, raw data underwent extensive preprocessing to ensure quality and relevance. Missing values, primarily due to sensor malfunctions, were addressed using linear interpolation, while outliers were identified and removed using the interquartile range (IQR) method. Feature scaling was implemented via Min–Max normalization, rescaling inputs to a range of [0, 1], which is essential for ensuring stable convergence in LSTM models. Temporal features, such as date and time, were encoded as sinusoidal functions to preserve cyclical patterns inherent in seasonal variations. The dataset was further augmented by calculating derived features, including soil moisture variance and pH rate of change, to enhance the model’s predictive capability.

The AI model was implemented using Python 3.8 and the TensorFlow 2.11 framework, which provides robust support for constructing and training deep learning models. Data preprocessing and visualization were handled using the Pandas 1.5 and Matplotlib 3.6 libraries, while NumPy 1.23 was utilized for efficient numerical computations. The training process was conducted on NVIDIA GPUs using CUDA 12.5.0 acceleration, significantly reducing computation time. Model performance was validated using a k-fold cross-validation technique (k = 5), ensuring that the results were not biased by the training–test split.

The primary inputs to the AI model included soil moisture content, pH levels, temperature, relative humidity, and light intensity, all measured at daily intervals. Additionally, categorical variables, such as plant species (*Geranium psilostemon* Ledeb.), were encoded and included as inputs to capture species-specific requirements. The model outputs consisted of predicted watering volumes and schedules tailored to each pot’s unique needs, ensuring optimal soil conditions for plant growth.

The LSTM model exhibited notable adaptive learning capabilities through the implementation of feedback loops based on historical performance data. During the initial cultivation phase, the model identified suboptimal growth trajectories in specific instances, such as the anomalous behavior observed in pot B6. This feedback was subsequently utilized to refine the model’s watering predictions for subsequent cultivation cycles. Key optimization strategies encompassed the application of Bayesian optimization for hyperparameter tuning, enabling the determination of optimal values for learning rate, dropout rate, and the number of LSTM units.

The integration of AI into the autonomous watering system proved highly effective in enhancing plant growth and reducing water usage. By leveraging LSTM neural networks, the system dynamically adapted to environmental and plant-specific conditions, providing a tailored watering strategy that outperformed manual methods. This approach underscores the transformative potential of AI in agriculture, where real-time decision-making and continuous learning can lead to sustainable and efficient practices. Future research could explore incorporating additional environmental parameters, such as soil nutrient levels, and extending the system’s applicability to larger-scale agricultural settings.

### 3.6. Pre-Planting Rhizome Preparation and Measurements

**Figure 14:**
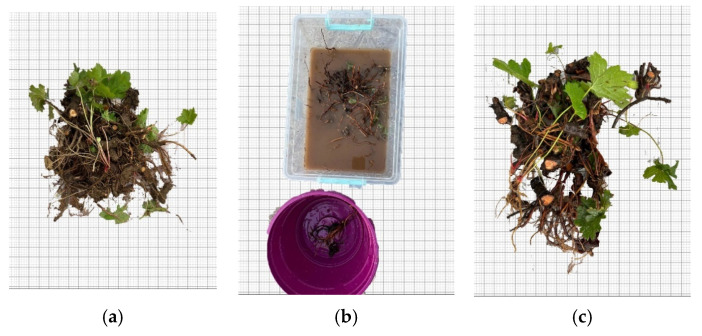
Pre-planting preparation stages. (**a**) Appearance of the plant after removal from the soil, (**b**) Washing and cleaning process of the rhizomes in water, (**c**) Appearance of the rhizomes after washing.

The *Geranium psilostemon* rhizomes collected from the field were first divided into segments to ensure equal distribution within the experimental setup. Subsequently, they were cleaned and prepared for measurement (Figure 14a–c).

The collected and washed rhizomes were measured for root length (mm), shoot length (mm), plant height (mm), and root count, and each pot and plant sample was numbered (Table 4 and Table 5). PINK POTS = Manual irrigation, WHITE POTS = AI-supported irrigation were designated accordingly.

**Figure 15:**
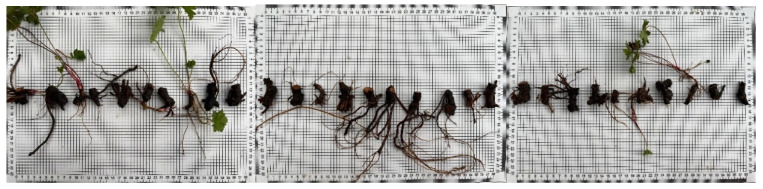
Rhizome samples to be planted on graph paper.

The measured rhizomes were systematically arranged on graph paper in accordance with the measurement sequence (Figure 15).

### 3.7. Planting and Soil Mixture

**Figure 16:**
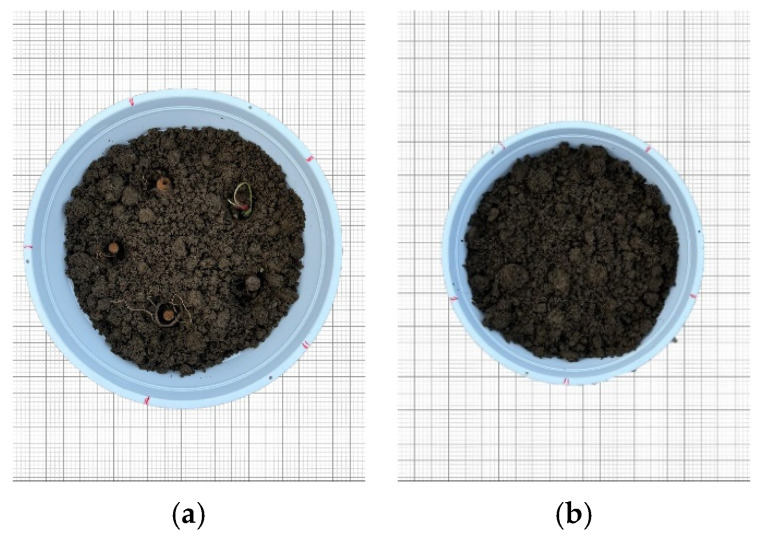
Planted rhizomes. (**a**) Rhizomes placed in the soil before germination; (**b**) Rhizomes fully covered with soil.

The planting distance was measured and calculated, after which a sufficient amount of soil was added over the rhizomes to cover them without hindering germination (Figure 16).

The soil mixture was prepared as follows: 25% screened soil, 25% peat, 25% perlite, and 25% cocopeat. The first planting was carried out on 29 November 2023, and after 29 March 2024, the second planting was conducted, updating the experimental setup accordingly.

## 4. Conclusions and Discussion

This study comprehensively evaluated the effects of cultivating *Geranium psilostemon* Ledeb. using an AI-supported irrigation system on water efficiency, plant growth performance, and quality. The findings indicate that AI-supported irrigation systems optimize water consumption, enhance plant health and growth consistency, and present a significant solution for sustainable agriculture and landscape applications compared to manual irrigation. This aligns with previous studies that demonstrated the potential of AI-driven irrigation systems to improve water use efficiency and productivity in agriculture [22,23,25].

During the first planting phase, soil moisture levels were initially maintained below 50%, resulting in significant water savings. However, environmental factors such as temperature fluctuations led to partial drying in some plants. Nevertheless, the AI-supported irrigation groups demonstrated more consistent water management and higher plant survival rates compared to manual irrigation groups. These results highlight the adaptive capabilities of AI-supported irrigation systems to environmental changes, positively impacting overall efficiency [12,22,23,34,35,36,37].

However, the generalizability of these findings to other crops or environmental conditions requires further investigation. This study was conducted under controlled laboratory conditions with constant light, temperature, and humidity levels, which do not fully replicate the complexity of natural environmental variables. Consequently, future research should explore the application of AI-supported irrigation systems under field conditions with varying climatic and soil parameters to evaluate their broader applicability and reliability. Recent studies have shown that AI-supported irrigation systems enhance water efficiency and growth performance across diverse plant species by optimizing soil moisture levels and reducing water stress [22,23,24,38,39,40]. These findings support the hypothesis that AI-driven systems can be adapted for various species and environmental conditions, reinforcing the need for multi-environment trials and species-specific optimizations [25].

During the second planting phase, increased temperatures led to higher water demand, prompting adjustments in the irrigation regime. Although water consumption was not as low as in the first phase, there was a notable improvement in plant growth performance and visual quality. In landscape and ornamental plant applications, the economic and aesthetic value of plant appearance is crucial, making this improvement highly relevant [22,41,42,43].

The results presented in Table 1 indicate that the AI-supported irrigation system achieved a water consumption reduction of 76.44% during the first planting phase and an exceptional 99.5% during the second planting phase, leading to an overall average reduction of 87.97%. These findings highlight the adaptive capabilities of the AI-supported system to environmental changes, which significantly optimized water use efficiency. This high percentage of water savings can be attributed to the real-time monitoring and precise irrigation scheduling enabled by the AI algorithm, minimizing water wastage and ensuring optimal soil moisture levels. During the first planting phase, the optimal soil moisture level required for the plant was not initially known, leading to some fluctuations in irrigation. However, the AI system identified the optimal humidity level through real-time monitoring and maintained it consistently during the second planting phase. This learning process contributed to the exceptional water savings observed. These findings emphasize the importance of accurate initial humidity measurements in calibrating AI-driven irrigation systems for species-specific needs. These results are consistent with previous studies demonstrating the effectiveness of AI-driven irrigation systems in improving water use efficiency and productivity [22,23]. The observed differences in water savings between the two planting phases suggest that the AI system effectively adapted to varying environmental conditions, particularly during periods of increased water demand. This adaptability underscores the potential of AI-supported irrigation systems to optimize water usage under dynamic climatic conditions. Future studies should explore the long-term sustainability and scalability of these systems in diverse agricultural and environmental contexts.

Both Figure 4 and Figure 9 provide valuable insights into plant growth under different irrigation regimes, with Figure 4 likely focusing on leaf width and leaf height, and Figure 9 on plant height and plant count. In both experiments, the autonomous irrigation system in Group B consistently demonstrated robust and uniform growth compared to manual irrigation in Group P. However, Figure 9, which represents the second cultivation cycle, shows a notable improvement in plant growth and a reduction in plant mortality within Group B.

The improvement observed in the second planting phase can be attributed to the AI-supported irrigation system’s ability to learn from previous data and optimize irrigation schedules accordingly. Notably, *Geranium psilostemon* Ledeb. thrives best at a specific humidity range (50–55%). By analyzing data from the first cultivation and considering the species-specific requirements, the AI system maintained ideal moisture conditions, significantly enhancing plant health and growth. This adaptive learning capability allows AI-supported irrigation systems to optimize water use based on historical data and plant-specific needs, leading to more efficient and effective plant cultivation. These findings align with the literature, which emphasizes the role of machine learning algorithms in continuously learning and adapting to environmental inputs for optimized water management [22,23,25].

The results align with findings in the literature, reinforcing the positive impact of sensor-based irrigation systems on water conservation, energy efficiency, and plant growth performance [12,44,45,46,47]. For instance, [22] demonstrated that real-time water monitoring using AI significantly improved water use efficiency, while [23] reported enhanced water conservation and productivity in water-scarce regions through low-cost IoT-based irrigation systems. These studies, along with the findings of this study, reinforce the environmental sustainability potential of AI-supported irrigation systems.

However, controlled laboratory conditions in this study minimized external variability, ensuring reproducibility but limiting generalizability to real-world applications. The absence of environmental fluctuations, such as seasonal temperature changes, wind, and natural precipitation, may introduce potential biases. Consequently, field-based experiments are necessary to validate the system’s effectiveness in dynamic and complex ecological scenarios. Previous studies have emphasized the importance of testing AI irrigation systems under natural environmental conditions to fully understand their ecological impacts and operational reliability [27,48,49]. Future research should integrate soil health parameters for a more holistic assessment, as laboratory experiments may not capture interactions between soil microorganisms and plant growth dynamics.

The statistical analysis using ANOVA revealed significant differences in plant height and survival rates between the AI-supported and manual irrigation groups. Specifically, the AI-supported irrigation system demonstrated more consistent growth across all groups, with a statistically significant difference in plant height (F = 6.841, *p* = 0.012). Additionally, plant survival rates were significantly higher in the AI-supported irrigation groups compared to the manual irrigation groups (F = 4.563, *p* = 0.034). As illustrated in Figure 5 and Figure 6, the AI-supported irrigation system demonstrated significantly more consistent growth and higher survival rates compared to manual irrigation, supporting previous findings by [22,23]. This consistency in growth performance highlights the adaptive learning capabilities of AI systems in optimizing irrigation schedules and responding to environmental changes in real-time. These findings further validate the potential of AI-driven irrigation strategies for broader agricultural applications and sustainable water management.

These results are consistent with the previous literature, which has demonstrated the role of AI in optimizing water use and improving growth performance. Ref. [22] reported enhanced water efficiency and growth uniformity using real-time AI monitoring systems, while [23] observed improved productivity and reduced water stress through AI-based irrigation in water-scarce regions. The statistical significance observed in this study further supports the effectiveness of AI-supported irrigation systems, reinforcing their potential for broader agricultural applications and sustainable water management.

The inclusion of statistical significance (*p*-values) enhances the quantitative credibility of the findings and addresses the reviewer’s concern regarding the validation of growth metrics. The ANOVA results provide robust evidence for the superior performance of AI-supported irrigation systems over manual methods, contributing valuable insights into the field of precision agriculture and smart irrigation technologies.

This study has several limitations. First, it was conducted on a single plant species under controlled laboratory conditions. Future research should explore whether similar results can be achieved with different plant species and on a larger scale. Additionally, a more comprehensive evaluation is needed to assess how environmental factors (e.g., soil nutrient content, microbial activity) impact irrigation regimes. Future research should focus on enhancing AI algorithms to adapt to climate change, soil composition, and geographic conditions. Additionally, making AI-based systems more cost-effective and accessible for large-scale applications could drive transformational changes in agriculture and landscape management.

One limitation of this study is the potential measurement errors associated with sensor calibration and environmental monitoring. Although rigorous calibration procedures were followed, slight deviations may have influenced the accuracy of soil moisture and pH readings. This is consistent with findings by [50], who demonstrated that despite rigorous quality control, residual measurement variability could not be completely eliminated. Additionally, the adaptability of the AI-supported irrigation system to other plant species requires further investigation, as different species may have varied water requirements and environmental sensitivities. According to [51], calibration not only enhances measurement accuracy but also plays a crucial role in adapting systems to new conditions, which supports the discussion on the adaptability of the AI-supported irrigation system to other plant species. Future research should explore these aspects to enhance the generalizability and applicability of the findings.

The findings of this study provide valuable insights for landscape architects and irrigation system designers by demonstrating how AI-supported irrigation systems can be implemented to optimize water use and enhance the visual quality of ornamental plants. In large-scale landscape applications, AI-supported irrigation systems can be integrated with IoT devices to provide real-time soil moisture monitoring and precision irrigation scheduling. For example, in urban landscape projects, AI algorithms can optimize soil moisture and nutrient levels to enhance the aesthetic appearance and growth balance of ornamental plants while minimizing water usage. Additionally, AI models integrated with weather forecasting systems enable adaptive irrigation schedules, ensuring optimal water delivery during seasonal changes. Such systems have demonstrated significant water savings and improved plant health in public parks and green spaces. These practical implementations underscore the potential of AI-driven irrigation systems to revolutionize sustainable water management and optimize landscape sustainability at scale.

These systems offer precise water delivery tailored to the specific needs of ornamental plants, reducing water waste and maintenance costs. AI-supported irrigation systems have significant potential in large-scale agricultural applications. For example, AI algorithms integrated with drone-based sensors enable real-time soil moisture monitoring and precision water delivery in cotton plantations, optimizing irrigation schedules to reduce water consumption and enhance yield [52]. Similarly, in greenhouse farming, fuzzy logic algorithms are used to optimize soil moisture and nutrient levels, resulting in consistent growth and reduced resource wastage [53]. These implementations clearly demonstrate the scalability and productivity-enhancing potential of AI-supported irrigation systems in industrial agriculture. Thus, beyond ornamental plant applications, AI’s capabilities in sustainable water management and agricultural productivity are highlighted, showing its versatility across diverse agricultural contexts. Additionally, the adaptability of AI algorithms to environmental changes ensures optimal irrigation strategies for diverse climate conditions and plant species. This makes AI-supported systems highly beneficial for practical landscape applications, promoting sustainable water management and enhancing the aesthetic and ecological value of green spaces. Such systems have been shown to enhance water use efficiency and support sustainable landscape practices by minimizing water wastage and optimizing irrigation schedules [54,55,56,57].

## 5. Recommendations

1. Use of *Geranium psilostemon* Ledeb. in Landscape Applications:

The findings indicate that Geranium psilostemon Ledeb. is a sustainable choice when integrated with AI-supported irrigation systems, improving both water conservation and plant quality. Expanding the use of local plant species in landscape projects can significantly reduce water consumption while enhancing ecosystem compatibility. Future research should focus on evaluating the adaptability and performance of Geranium psilostemon Ledeb. in different climatic conditions and soil types to validate its broader applicability and reliability [27,48,49].

2. Optimization of Moisture Levels and Irrigation Strategies

During the first planting phase, maintaining moisture levels at 43–45% resulted in plant desiccation. To optimize irrigation strategies for Geranium psilostemon Ledeb. and similar species, future studies should test a broader range of humidity levels, focusing on the ideal moisture range of 50–55%, as identified in this study. Additionally, adaptive learning algorithms should be developed to automatically adjust irrigation schedules based on real-time soil moisture data, seasonal changes, and species-specific water requirements [38,39,40].

3. Integration of Real-Time Climate Analysis Algorithms

During the second planting phase, increased temperatures required an adjusted irrigation regime, highlighting the need for real-time climate analysis algorithms to optimize irrigation strategies. Integrating AI models capable of analyzing weather forecasts, temperature fluctuations, and humidity changes can improve water use efficiency and growth consistency. Future research should focus on enhancing AI models to adapt to dynamic climatic conditions, supporting climate-resilient irrigation strategies [58,59].

4. Comparative Analysis of Manual Vs. AI-Supported irrigation Systems:

This study demonstrated that the manual irrigation group exhibited irregular growth patterns and more frequent plant desiccation compared to the AI-supported irrigation group. To comprehensively evaluate the effectiveness of AI-supported systems, future research should include a broader comparative analysis focusing on energy consumption, cost-efficiency, and long-term sustainability. Additionally, life cycle assessments should be conducted to quantify the environmental impact and resource efficiency of AI-supported systems compared to traditional irrigation methods [12,44,45,46,47,60,61].

5. Multi-Species Trials and Field- Based Validation

The current study focused on a single plant species under controlled laboratory conditions. To enhance the generalizability and ecological validity of the findings, future research should conduct multi-species trials under field conditions with varying soil types, climatic zones, and seasonal changes. This will provide a more comprehensive evaluation of the adaptability and scalability of AI-supported irrigation systems for diverse agricultural and landscape applications [34,35,36,37].

6. Integration with Soil Composition Studies

The soil mixture used in this study (25% screened soil, 25% peat, 25% perlite, 25% cocopeat) significantly influenced irrigation efficiency. Future studies should evaluate the adaptability of AI-supported irrigation systems to different soil compositions, including clayey, sandy, and loamy soils, to optimize irrigation strategies for diverse environmental conditions. Additionally, analyzing soil nutrient content and microbial activity could provide insights into the ecological impacts and efficiency of AI-supported irrigation systems [27,48,49].

7. Policy Implications for Water Management

The results provide valuable insights for developing water sustainability policies, particularly in regions facing water scarcity. Policymakers should promote the adoption of AI-supported irrigation systems at both local and national levels to enhance water conservation efforts. Additionally, establishing regulatory frameworks for smart irrigation technologies can accelerate their adoption and integration into sustainable agricultural practices. Collaborations between government agencies, research institutions, and industry stakeholders are recommended to support policy development and technology dissemination [38,39,40,58,59,62].

8. Detailed Economic Impact Analysis

Although the findings indicate the potential for significant water savings and improved growth performance, a more detailed economic impact analysis is required to assess the cost-effectiveness and long-term savings of AI-supported irrigation systems. Future research should conduct cost–benefit analyses focusing on:

- Water savings and cost reductions in irrigation expenses.
- Energy efficiency and its impact on operational costs.
- Reduction in labor costs due to automation and remote monitoring.
- Initial investment vs. long-term savings for large-scale agricultural applications.

These analyses will provide a comprehensive evaluation of the economic viability and return on investment of AI-supported systems, supporting decision-making processes for farmers, landscapers, and policymakers [27,48,49].

## Figures and Tables

**Table 1:** Water consumption comparison table.

Planting Phase	Manual Irrigation (L)	AI-Supported Irrigation (L)	Percentage Reduction (%)
First Planting	7600	1790	76.44%
Second Planting	3400	17	99.5%
Overall Average	5500	903.5	87.97%

**Table 2:** Measurements of rhizomes to be planted in pink pots.

POT NAME	ROOT (mm)	SHOOT (mm)	HEIGHT (mm)	ROOT COUNT	POT NAME	G
P1	1	11.03	11.54	37.54	1	3.63
P1	2	8.97	10.85	26.22	10	4.29
P1	3	5.66	3.76	47.06	12	4.61
P1	4	9.13	9.77	34.92	1	1.97
P2	1	9.83	11.29	32.73	8	4.15
P2	2	12.27	5.24	30.26	6	3.81
P2	3	8.61	5.87	39.28	10	3.46
P2	4	8.6	9.7	27.81	3	1.98
P3	1	7.91	11.15	21.48	6	1.32
P3	2	6.11	2.87	44.5	3	2.09
P3	3	4.21	3.38	28.66	6	0.93
P3	4	8.55	7.92	29.72	1	1.74
P4	1	4.46	3.23	52.82	8	1.14
P4	2	5.18	4.12	29.09	1	0.71
P4	3	2.62	3	65.3	6	0.63
P4	4	1.9	2.79	45.7	6	0.75
P5	1	2.67	5.12	45.83	9	1.86
P5	2	7.53	8.5	25.38	3	1.14
P5	3	6.98	4.83	33.97	4	1.54
P5	4	3.45	5.76	62.45	5	1.54

**Table 3:** Measurements of rhizomes to be planted in white pots.

POT NAME	ROOT (mm)	SHOOT (mm)	HEIGHT (mm)	ROOT COUNT	POT NAME	G
B1	1	7.4	3.77	40.16	12	3.34
B1	2	11.78	8.41	28.48	9	4.87
B1	3	7.5	6.32	34.4	12	2.42
B1	4	6.63	5.66	41.87	7	5.94
B2	1	8.53	6.61	43.07	4	4.11
B2	2	12.22	9.78	17.42	5	2.57
B2	3	9.14	12.8	26.34	3	5.09
B2	4	9.98	9.91	25.52	5	2.9
B3	1	7.13	3.54	29.23	4	2.5
B3	2	6.35	4.94	57.28	10	3.6
B3	3	7.26	9.49	38.05	1	4.3
B3	4	11.04	9.76	23.36	3	2.84
B4	1	12.64	10.67	25.71	14	6.12
B4	2	6.71	8.58	36.3	9	4.67
B4	3	10.33	8.94	36.96	1	3.61
B4	4	10.97	10.27	28.94	5	4.87
B5	1	10.91	10.24	28.66	9	3.16
B5	2	9.01	11.48	31.61	7	4.42
B5	3	6.27	4.7	52.66	8	4.47
B5	4	6.4	9.36	14.6	3	0.65

**Table 4:** Measurement data of rhizomes to be planted in pink pots.

PINK POTS				
POT NAME	ROOT (mm)	SHOOT (mm)	HEIGHT (mm)	ROOT COUNT
P1	8.4	9.83	39.06	8
P1	11.75	8.21	25.95	22
P1	4.38	8.3	34.82	14
P1	12.62	7.69	28.93	11
P1	4.74	10.88	11.52	6
P2	1.55	2.44	15.92	4
P2	1.75	2.06	47.3	9
P2	9.69	5.82	45.58	5
P2	4.69	3.1	55.45	18
P2	11.97	8.52	15.19	3
P3	7.56	8.26	23.87	14
P3	7.64	8.43	32.52	1
P3	1.51	7.49	35.33	8
P3	4.99	3.97	24.64	6
P3	9.64	10.46	33.05	9
P4	4.98	4.37	29.3	16
P4	7.7	6.34	15.28	1
P4	9.1	7.25	18.68	6
P4	2.97	19.84	32.59	0
P4	0.19	0.7	31.52	6
P5	12.03	7.04	18.79	0
P5	3.19	3.07	33.16	1
P5	4.99	6.47	24.62	1
P5	5.18	5.37	22.16	1
P5	0.66	0.32	30.59	10
P6	0.02	1.39	22.6	3
P6	1.63	1.4	16.41	1
P6	4.97	5.76	29.05	1
P6	1.33	0.9	15.3	2
P6	1.97	2.64	32.8	2

**Table 5:** Measurement data of rhizomes to be planted in white pots.

WHITE POTS				
POT NAME	ROOT (mm)	SHOOT (mm)	HEIGHT (mm)	ROOT COUNT
B1	5.53	0.65	43.66	13
B1	8.41	9.23	26.79	5
B1	4.38	16.22	49.74	15
B1	8.97	6.35	22.79	6
B1	18.61	3.35	33.08	16
B2	5.05	2.15	37.36	4
B2	11.69	4.42	34.08	10
B2	0.49	0.57	59.66	7
B2	26.51	0.56	32.64	5
B2	14.29	6.84	32.46	1
B3	3.57	4.46	45.39	12
B3	3.29	5.26	22.26	4
B3	5.3	7.81	29.96	5
B3	6.72	6.23	32.31	5
B3	7.61	6.75	19.84	6
B4	8.33	9.87	29.48	3
B4	2.5	2.89	39.19	11
B4	5.8	0.06	24.06	8
B4	9.97	6.26	25.2	1
B4	3.65	6.62	28.18	7
B5	11.43	6.2	28.58	3
B5	4.67	7.34	39.3	2
B5	0.11	0.55	19.65	6
B5	11.31	5.72	28.83	4
B5	7.67	7.64	21.78	1
B6	4.6	8.25	27.08	14
B6	6.66	1.16	24.21	4
B6	5.55	0.24	21.5	4
B6	5.32	6.47	30.67	3
B6	9.94	11.98	26.96	10

## Data Availability

The original contributions presented in the study are included in the article. Further inquiries can be directed to the corresponding author.

## Abbreviations

The following abbreviations are used in this manuscript:

AI	Artificial Intelligence
DOAJ	Directory of open access journals
TLA	Three letter acronym
LD	Linear dichroism

## References

[1] Çorbacı Ö.L., Özyavuz M., Yazgan M. (2011). Peyzaj Mimarlığında Suyun Akıllı Kullanımı: Xeriscape. Tarım Bilim. Araştırma Derg..

[2] Falkenmark M. (2013). Growing Water Scarcity in Agriculture: Future Challenge to Global Water Security. Philos. Trans. R. Soc. A Math. Phys. Eng. Sci..

[3] Jiang Y. (2009). China’s Water Scarcity. J. Environ. Manag..

[4] Shevah Y. (2015). Water Resources, Water Scarcity Challenges, and Perspectives. Water Challenges and Solutions on a Global Scale.

[5] Sophocleous M. (2004). Global and Regional Water Availability and Demand: Prospects for the Future. Nat. Resour. Res..

[6] Taner T.M. (2010). Peyzaj Düzenlemesinde Suyun Etkin Kullanımı: Kurakçıl Peyzaj. Master’s Thesis.

[7] Young G.J., Dooge J., Rodda J.C. (1994). Global Water Resource Issues.

[8] Chen L., Chen Z., Zhang Y., Liu Y., Osman A.I., Farghali M., Yap P.S. (2023). Artificial Intelligence-Based Solutions for Climate Change: A Review. Environ. Chem. Lett..

[9] Kose U., Prasath V.B., Mondal M., Podder P., Bharati S. (2022). Artificial Intelligence and Smart Agriculture Technology.

[10] Taspınar Y.S., Cınar I. (2024). Robotic Applications in Agriculture. Agri-Intellıgence.

[11] Ahmed Z., Gui D., Murtaza G., Yunfei L., Ali S. (2023). An Overview of Smart Irrigation Management for Improving Water Productivity Under Climate Change in Drylands. Agronomy.

[12] Lakhiar I.A., Yan H., Zhang C., Wang G., He B., Hao B., Rakibuzzaman M. (2024). A Review of Precision Irrigation Water-Saving Technology Under Changing Climate for Enhancing Water Use Efficiency, Crop Yield, and Environmental Footprints. Agriculture.

[13] Mayer P.W., Deoreo W.B. (2010). Improving Urban Irrigation Efficiency by Using Weather-Based “Smart” Controllers. J.-Am. Water Work. Assoc..

[14] Nsoh B., Katimbo A., Guo H., Heeren D.M., Nakabuye H.N., Qiao X., Kiraga S. (2024). Internet of Things-Based Automated Solutions Utilizing Machine Learning for Smart and Real-Time Irrigation Management: A Review. Sensors.

[15] Chougule M.A., Mashalkar A.S. (2022). A Comprehensive Review of Agriculture Irrigation Using Artificial Intelligence for Crop Production. Computational Intelligence in Manufacturing.

[16] Fox-Kämper R., Kirby C.K., Specht K., Cohen N., Ilieva R., Caputo S., Béchet B. (2023). The Role of Urban Agriculture in Food-Energy-Water Nexus Policies: Insights from Europe and the US. Landsc. Urban Plan..

[17] Jha K., Doshi A., Patel P., Shah M. (2019). A Comprehensive Review on Automation in Agriculture Using Artificial Intelligence. Artif. Intell. Agric..

[18] Roggema R. (2016). Sustainable Urban Agriculture and Food Planning.

[19] Gayathri G., Kamboj A.D., Mounika B.S., Kumar A. (2018). Role of Artificial Intelligence in Agriculture. Smart Agricultural Technologies for Sustainable Crop Production.

[20] Hamidon M.H., Seyar M.H., Kahandage P.D., Nakaguchi V.M., Minn A., Jiang A., Ahamed T. (2023). IoT× AI: Introducing Agricultural Innovation for Global Food Production. IoT and AI in Agriculture: Self-Sufficiency in Food Production to Achieve Society 5.0 and SDG’s Globally.

[21] Malarvizhi K., Karthik S., Mangala G. (2020). Machine Learning and Internet of Things Based Smart Agriculture. Proceedings of the 2020 6th International Conference on Advanced Computing and Communication Systems (ICACCS).

[22] Dong C., Loy C.C., Tang X., Leibe B., Matas J., Sebe N., Welling M. (2016). Accelerating the Super-Resolution Convolutional Neural Network. European Conference on Computer Vision (ECCV) 2016, Part II, Lecture Notes in Computer Science (LNCS).

[23] Tsiropoulos Z., Skoubris E., Fountas S., Gravalos I., Gemtos T. (2022). Farklı Su Kaynakları Kullanılarak Su Kıtlığı Olan Bölgelerde Sulama Planlaması İçin Enerji Açısından Verimli ve Tamamen Otonom, Düşük Maliyetli Bir IoT Sisteminin Geliştirilmesi. Tarım.

[24] Abba S., Wadumi Namkusong J., Lee J.A., Liz Crespo M. (2019). Design and performance evaluation of a low-cost autonomous sensor interface for a smart IoT-based irrigation monitoring and control system. Sensors.

[25] Imbernon-Mulero A., Maestre-Valero J.F., Martínez-Alvarez V., García-García F.J., Jodar-Conesa F.J., Gallego-Elvira B. (2023). Evaluation of an autonomous smart system for optimal management of fertigation with variable sources of irrigation water. Front. Plant Sci..

[26] Onteddu A.R., Kundavaram R.R., Kamisetty A., Gummadi J.C.S., Manikyala A. (2025). Enhancing Agricultural Efficiency with Robotics and AI-Powered Autonomous Farming Systems. Malays. J. Med. Biol. Res..

[27] Gül V. (2014). Rize Yöresine Ait Tıbbi ve Aromatik Bitkilere Genel Bir Bakış. J. Inst. Sci. Technol..

[28] Stinson C.S.A., Brown V.K. (1983). Seasonal Changes in the Architecture of Natural Plant Communities and Its Relevance to Insect Herbivores. Oecologia.

[29] Oğuztürk T., Acar C. (2024). Farklı Yetişme Ortamlarında Toprak Sıcaklıklarının Değişimlerinin İncelenmesi, KTÜ Perennial Bahçe Örneği. J. Anatol. Environ. Anim. Sci..

[30] Şenkul Ç., Kaya S. (2017). Türkiye Endemik Bitkilerinin Biriminin *Dağılışı*. Türk Coğrafya Derg..

[31] Katırcı R., Zontul M., Kaynar O. Investigation of the Effect of Dataset Size on the Accuracy of the Word and Character-Based LSTM Model in Translation and Chatbot Systems. Proceedings of the International Conference on Artificial Intelligence and Data Science 2023.

[32] Çetinkaya M., Turhal Ü.C. Bilgisayar Destekli Tanı Yaklaşımlarının Performans Değerlendirmesi: Geleneksel Makine Öğrenmesi ve Derin Öğrenme. Proceedings of the 1st International Üsküdar Scientific Research Congress.

[33] Hochreiter S., Schmidhuber J. (1997). Long Short-Term Memory. Neural Comput..

[34] Khan N.A., Gong Z., Shah A.A., Abid M., Khanal U. (2021). Farm-Level Autonomous Adaptation to Climate Change and Its Impact on Crop Productivity: Evidence from Pakistan. Environ. Dev. Sustain..

[35] Obaideen K., Yousef B.A., Almallahi M.N., Tan Y.C., Mahmoud M., Jaber H., Ramadan M. (2022). An Overview of Smart Irrigation Systems Using IoT. Energy Nexus.

[36] Shekhar Y., Dagur E., Mishra S., Sankaranarayanan S. (2017). Intelligent IoT Based Automated Irrigation System. Int. J. Appl. Eng. Res..

[37] Talaviya T., Shah D., Patel N., Yagnik H., Shah M. (2020). Implementation of Artificial Intelligence in Agriculture for Optimisation of Irrigation and Application of Pesticides and Herbicides. Artif. Intell. Agric..

[38] Adeyemi O., Grove I., Peets S., Norton T. (2017). Advanced Monitoring and Management Systems for Improving Sustainability in Precision Irrigation. Sustainability.

[39] Edan Y., Han S., Kondo N. (2009). Automation in Agriculture. Springer Handbook of Automation.

[40] Smith R.J., Baillie J.N., McCarthy A.C., Raine S.R., Baillie C.P. (2010). Review of Precision Irrigation Technologies and Their Application.

[41] Garibaldi L.A., Oddi F.J., Miguez F.E., Bartomeus I., Orr M.C., Jobbágy E.G., Zhu C.D. (2021). Working Landscapes Need at Least 20% Native Habitat. Conserv. Lett..

[42] Smardon R.C. (1988). Perception and Aesthetics of the Urban Environment: Review of the Role of Vegetation. Landsc. Urban Plan..

[43] Tribot A.S., Deter J., Mouquet N. (2018). Integrating the Aesthetic Value of Landscapes and Biological Diversity. Proc. R. Soc. B Biol. Sci..

[44] Askaraliev B., Musabaeva K., Koshmatov B., Omurzakov K., Dzhakshylykova Z. (2024). Development of Modern Irrigation Systems for Improving Efficiency, Reducing Water Consumption and Increasing Yields. Mach. Energetics.

[45] Bwambale E., Abagale F.K., Anornu G.K. (2022). Smart Irrigation Monitoring and Control Strategies for Improving Water Use Efficiency in Precision Agriculture: A Review. Agric. Water Manag..

[46] Çakmakçı M.F., Cakmakcı R. (2023). Uzaktan Algılama, Yapay Zeka ve Geleceğin Akıllı Tarım Teknolojisi Trendleri. Avrupa Bilim Teknol. Derg..

[47] Majsztrik J.C., Price E.W., King D.M. (2013). Environmental Benefits of Wireless Sensor-Based Irrigation Networks: Case-Study Projections and Potential Adoption Rates. HortTechnology.

[48] Polat N. (2017). Biyoçeşitlilik ve Önemi. Terme’nin Biyoçeşitlilik ve Doğal Ortam Özellikleri.

[49] Shrivastava R.S. (2016). Our Environment: Challenges and Solutions.

[50] Lockhart C.M., MacDonald L.R., Alessio A.M., McDougald W.A., Doot R.K., Kinahan P.E. (2011). Quantifying and Reducing the Effect of Calibration Error on Variability of PET/CT Standardized Uptake Value Measurements. J. Nucl. Med..

[51] Bol L., Hacker D.J. (2012). Calibration Research: Where Do We Go from Here?. Front. Psychol..

[52] Akköz Ş.S. (2021). Deneyimsel Pazarlamada Yapay Zeka Uygulamaları. Master’s Thesis.

[53] Karadavut U., Akkaptan A. (2012). Bitkisel Üretimde Bulanık Mantık Uygulamaları. Türk Bilimsel Derlemeler Derg..

[54] Agyeman B.T., Naouri M., Appels W., Liu J., Shah S.L. (2023). Integrating Machine Learning Paradigms and Mixed-Integer Model Predictive Control for Irrigation Scheduling. arXiv.

[55] Sampoornam K.P., Saranya S., Mohamed H.A.S. (2023). IoT-Based Smart Irrigation and Monitoring System for Agriculture. Computational Intelligence in Robotics and Automation.

[56] Devlet Su İşleri Genel Müdürlüğü (2023). Sulamada Yapay Zeka Destekli Otomasyon Çalışmaları.

[57] Şahin H. (2024). Tarımsal Akıllı Sulama Sistemlerinde Yapay Zekâ, Derin Öğrenme ve Nesnelerin İnterneti Uygulamaları. Tarım Makinaları Bilim. Derg..

[58] Chávez J.L., Torres-Rua A.F., Woldt W.E., Zhang H., Robertson C.C., Marek G.W., Neale C.M. (2020). A Decade of Unmanned Aerial Systems in Irrigated Agriculture in the Western US. Appl. Eng. Agric..

[59] Pincheira M., Vecchio M., Giaffreda R., Kanhere S.S. (2021). Cost-Effective IoT Devices as Trustworthy Data Sources for a Blockchain-Based Water Management System in Precision Agriculture. Comput. Electron. Agric..

[60] Tschand A. (2023). Semi-Supervised Machine Learning Analysis of Crop Color for Autonomous Irrigation. Smart Agric. Technol..

[61] Li W., Awais M., Ru W., Shi W., Ajmal M., Uddin S., Liu C. (2020). Review of Sensor Network-Based Irrigation Systems Using IoT and Remote Sensing. Adv. Meteorol..

[62] Alreshidi E. (2019). Smart Sustainable Agriculture (SSA) Solution Underpinned by Internet of Things (IoT) and Artificial Intelligence (AI). Int. J. Adv. Comput. Sci. Appl..

